# Tissue-Specific Transcriptome Analysis Reveals Candidate Genes for Terpenoid and Phenylpropanoid Metabolism in the Medicinal Plant *Ferula assafoetida*

**DOI:** 10.1534/g3.118.200852

**Published:** 2019-01-24

**Authors:** Hajar Amini, Mohammad Reza Naghavi, Tong Shen, Yanhong Wang, Jaber Nasiri, Ikhlas A. Khan, Oliver Fiehn, Philipp Zerbe, Julin N. Maloof

**Affiliations:** * Department of Plant Biology, University of California, Davis, CA 95616; ‡ West Coast Metabolomics Center, University of California, Davis, CA 95616; † Department of Agronomy and Plant Breeding, Agricultural and Natural Resources College, University of Tehran, Iran, 77871-31587; § National Center for Natural Products Research, Research Institute of Pharmaceutical Sciences, University of Mississippi, Oxford, MS 38677; ** Department of Biochemistry, Faculty of Sciences, King Abdulaziz University, Jeddah, Saudi-Arabia

**Keywords:** RNAseq, coumarin-type phenylpropanoids, terpenoid, medicinal plant

## Abstract

*Ferula assafoetida* is a medicinal plant of the Apiaceae family that has traditionally been used for its therapeutic value. Particularly, terpenoid and phenylpropanoid metabolites, major components of the root-derived oleo-gum-resin, exhibit anti-inflammatory and cytotoxic activities, thus offering a resource for potential therapeutic lead compounds. However, genes and enzymes for terpenoid and coumarin-type phenylpropanoid metabolism have thus far remained uncharacterized in *F. assafoetida*. Comparative *de novo* transcriptome analysis of roots, leaves, stems, and flowers was combined with computational annotation to identify candidate genes with probable roles in terpenoid and coumarin biosynthesis. Gene network analysis showed a high abundance of predicted terpenoid- and phenylpropanoid-metabolic pathway genes in flowers. These findings offer a deeper insight into natural product biosynthesis in *F. assafoetida* and provide genomic resources for exploiting the medicinal potential of this rare plant.

*Ferula assafoetida* L. (Apiaceae) is a herbaceous, monoecious and perennial plant indigenous to Kashmir, Afghanistan, and Iran (Iranshahy and Iranshahi 2011). *F. assafoetida* is predominantly valued for its medicinal uses as an important source of oleo-gum-resin, called asafoetida, that is obtained from the exudates of the tap roots (Kavoosi and Rowshan 2013). Asafoetida has broad therapeutic properties, for example for the treatment of inflammations, neurological and digestive disorders, rheumatism, headache, arthritis, and dizziness (Iranshahy and Iranshahi 2011). The oleo-gum-resin consists of three main fractions, including resin (40–64%), gum (25%) and essential oil (10–17%) (Amalraj and Gopi 2017). Phenylpropanoids (especially coumarins-related compounds) and terpenoids are the major constituents of the resin (Amalraj and Gopi 2017), whereas the essential oil is mostly comprised of sulfur-containing compounds, as well as volatile mono- and sesqui-terpenoids (Divya *et al.* 2014). Among these metabolites, sesquiterpene coumarins are of particular importance, due to their extensive and promising biological properties (Iranshahi *et al.* 2008). Sesquiterpene coumarins, which contain a coumarin or 1-benzopyran-2-one group joint with a sesquiterpene scaffold, are almost exclusively found in the genus *Ferula* and accumulate mainly in the roots of the plant (Curini *et al.* 2006).

In plants, terpenoid metabolites are derived from two isomeric 5-carbon precursors, isopentenyl diphosphate (IPP) and dimethylallyl diphosphate (DMAPP), which are formed via the plastidial methyl-erythritol-5-phosphate (MEP) and the cytosolic mevalonate (MEV) pathway (Chen *et al.* 2011; Falara and Pichersky 2012). Prenyltransferase-catalyzed condensation of IPP and DMAPP units into prenyl diphosphate intermediates of different chain length provides central precursors that are further converted by species-specific enzymes families of terpene synthases (TPSs) and cytochrome P450 monooxygenases (P450s) to give rise to the chemical diversity of plant terpenoids (Chen *et al.* 2011; Pateraki *et al.* 2015) (Supplementary Figure S1A). In addition to terpenoids, flavonoids (especially luteolin) and coumarin-related compounds (especially umbelliferone) are enriched in *F. assafoetida* (Pangarova and Zapesochnaya 1973; Amalraj and Gopi 2017). These metabolites are derived from p-coumaroyl-CoA formed via the plastidial shikimate pathway (Naoumkina *et al.* 2010; Vogt 2010). Branching from this core intermediate, distinct 2-oxoglutarate-dependent dioxygenases, feruloyl-CoA-6’-hydroxylases (F6’H) and coumaroyl-CoA-2’-hydroxylases (C2’H), facilitate the key steps in the biosynthesis of umbelliferone and other coumarins (Matsumoto *et al.* 2012; Vialart *et al.* 2012; Tohge *et al.* 2013). By contrast, flavonoid biosynthesis requires the conversion of p-coumaroyl-CoA by a chalcone synthase (CHS) and a chalcone isomerase (CHI), followed by various possible functional modifications of the resulting naringenin intermediate (Naoumkina *et al.* 2010; Vogt 2010) (Supplementary Figure S1B).

Rapid advances in genomics and biochemical technologies have enabled a deeper investigation of metabolic pathways in range of medicinal and other non-model plants (Xiao *et al.* 2013; Zerbe *et al.* 2013; Kitaoka *et al.* 2015; Wurtzel and Kutchan 2016). Among members of the Apiaceae, genomics-enabled gene discovery in carrot (*Daucus carota*) was utilized to identify flavonoid and isoprenoid pathway genes (Iorizzo *et al.* 2016) and enabled the functional characterization of two carrot terpene synthase genes, the sesquiterpene synthase *DcTPS1* and the monoterpene synthase *DcTPS2* (Yahyaa *et al.* 2015).

In this study, we employed comparative *de novo* transcriptome analyses and computational gene annotation to identify candidate genes with probable roles in the biosynthesis of bioactive terpenoid and coumarin-type phenylpropanoid metabolites in *F. assafoetida*.

## Material And Methods

### Plant material

Roots, stems, flowers, and leaves of *F. assafoetida* were collected from the Molla Ahmad Mountains, Isfahan province, Iran, at an altitude of 2250 meters (53°35′E and 32°15′N). Samples were collected from three separate plants, which were used as biological replicates. The identity of the harvested plants was verified by the Iranian Biological Resource Center (IBRC).

### Metabolite analysis

Roots, stems, flowers, and leaves were air-dried in the shade at room temperature. Terpenoid and sesquiterpene-coumarin compounds present in the oleo-gum-resin were extracted from the essential oil. In the context of this study, essential oils were prepared by grinding 20 grams of plant organs (roots, stems, flowers, and leaves) to a fine powder. Essential oils were then isolated through hydro-distillation for 5 hr, using a Clevenger type apparatus. The distilled oils were dried over anhydrous sodium sulfate and after filtration stored at 4°. Terpenoid analysis was then performed via GC-MS analysis as described in the Supplementary Methods. Product identification was achieved by comparison to reference mass spectra and retention indices (RI) available through the US National Institute of Standards and Technology (NIST, USA), WILEY 1996 Ed. mass spectral library, as well as an in-house library.

Umbelliprenin, umbelliferone, and luteolin were quantified in different organs and oleo-gum-resins of *F. assafoetida* by ultra-high-performance liquid chromatography-quadrupole time-of-flight mass spectrometry (UHPLC-QToF-MS) at the National Center for Natural Products Research at the University of Mississippi as outlined in the Supplementary Methods.

### RNAseq library preparation and pre-processing of short reads

RNA was extracted using BioZOL total RNA extraction kit (BioFlux, Japan) as detailed in the manufacturer’s instructions. For removing polyphenol and polysaccharide content in different organs, especially roots, an additional purification step was applied as follows: The resulting pellets were dissolved in Diethyl Pyrocarbonate (DEPC)-treated water and extracted once with phenol-chloroform (1:1) and then with chloroform. The aqueous solution was transferred into 2 new tubes, and 3M sodium acetate (pH 5.2) with 5M NaCl and 0.6 volume of cold isopropanol was added. This solution was mixed and then stored at -20° for one hour. Next, the solution was centrifuged at 13,000 rpm for 10 min at 4°. The pellet was collected after centrifugation by discarding the upper aqueous phase. The pellet was washed twice with 75% ethanol by re-suspending the pellet and centrifuging at 10,000 rpm for 10 min at 4° at each of the wash steps. The ethanol was allowed to evaporate, and the pellet was resuspended in 40 µl of DEPC-treated water. Furthermore, we removed any contaminating DNA using RNase-free DNase I (Thermo Fisher Scientific Inc). Quality and quantity of RNA were assessed by separation of RNA by gel electrophoresis on a 1% agarose gel, NanoDrop (ND-1000) and Agilent 2100 Bioanalyzer. RNA samples with RNA integrity number (RIN) values >8.0 were selected for constructing libraries. RNA libraries were obtained according to Breath Adapter Directional sequencing (BrAD-seq) protocol (Townsley *et al.* 2015) with shotgun (SHO) type strand-specific libraries for sending to the DNA Technologies Core at UC Davis. RNA sequencing was performed taking steps for mRNA Fragmentation, 3-prime Adapter Priming and cDNA Synthesis, 5-prime Duplex Breath Capture Adapter Addition (Strand Specific) and enrichment and adapter extension. The paired-end sequencing with read length of 150 bp was performed on an Illumina HiSeq 4000 platform by the DNA Technologies Core at the UC Davis Genome Center. Another set of RNA samples were sent to the Beijing Genomics Institute (BGI) and Novogene for library preparation and sequencing. After sequencing, raw reads were separated by barcode and filtered by quality using the HiSeq 4000 software CASAVA V1.8. The read quality before and after quality control with Trimmomatic was tested with FastQC quality assessment (http://www.bioinformatics.babraham.ac.uk/projects/fastqc/) and results were collected from all samples into a single report for easy comparison with MultiQC (Ewels *et al.* 2016). The parameters used in Trimmomatic V0.33 (Bolger *et al.* 2014) for trimming and cropping the FASTQ data as well as removing adapters was set as follows: ILLUMINACLIP:2:30:10, LEADING:3, TRAILING:3, SLIDINGWINDOW:4:15, MINLEN:120. The summary of the RNAseq reads after trimming, cropping, and adapter removal is shown in Supplementary Table S2.

### De novo transcriptome assembly and evaluation

We utilized four different *de novo* transcriptome assembly pipelines to ensure that a high quality reference transcriptome was assembled (Supplemental Table S3). These four different *de novo* transcriptome assembly pipelines are described in more detail in the Supplementary Methods.

Reads from all organs were combined and assembled with Trinity v2.4.0 with kmer 25 (Grabherr *et al.* 2011) and Oases v0.2.06 with kmer 25, 31,37, 43, 49 (Zerbino and Birney 2008; Schulz *et al.* 2012). Drap v1.91 was applied as a post processing step to compact and correct each assembled transcriptome (Cabau *et al.* 2017). In addition to these methods, we used Khmer v2.0 tools (Crusoe *et al.* 2015) to apply variable kmer coverage abundance trimming to the reads prior to Trinity assembly. This reduces the computational cost of assembly without negatively affecting the quality of the assembly. Several different approaches were utilized to assess the quality of each assembled transcriptome. First, we investigated the length distribution of the transcripts produced by the different pipelines (Supplementary Figure S2A). Second, we mapped the reads from each sample to each assembled transcriptome with STAR 2.5.2b to determine the percentage of reads that mapped to each assembly (Supplementary Figure S2B) (Dobin *et al.* 2013). Finally, we assessed the completeness of each assembled transcriptome in terms of expected genes with BUSCO v3 (Simão *et al.* 2015; Waterhouse *et al.* 2018) and using “Plant set (Embryophyta *odb9*)” as a database of BUSCO group with 1440 genes (Supplementary Table S4).

### Transcriptome annotation

We first annotated the assembled transcriptome using the dammit tool (Scott 2016). In this analysis, dammit searched the well-known annotated protein databases, for example, Pfam (Finn *et al.* 2015), Rfam (Kalvari *et al.* 2017), OrthoDB (Zdobnov *et al.* 2016), BUSCO, and UniRef (Suzek *et al.* 2014) for significant matches against the *F. assafoetida* assembled transcriptomes using blast (E-value cutoff <1e^-5^). A large fraction of the assembled transcripts was assigned successfully to known annotated protein from other plants. Out of the 60,134 assembled transcripts, dammit was able to map 54,129 (>90%) to known genes using blast with an E-value <0.00001 (for each hit).

In addition, the most likely protein sequence was identified by using TransDecoder (http://transdecoder.github.io) to find the longest open reading frame. Furthermore, sequences were initially annotated by comparing *F. assafoetida* protein sequences against the Arabidopsis protein sequence database (TAIR10) (n = 35,386). We were able to successfully find a significant hit (E-value <1e^-10^) to 30,344 (>85%) of the Arabidopsis protein sequence, which covered 34,920 (58%) of the *F. assafoetida* assembled transcripts. We also used blastx to compare the assembled transcriptome against all RefSeq plants database (>10,710 plants) (Pruitt *et al.* 2006). Our observations indicated that a significant majority of our assembled contigs (>77%) had the best hit with *D. carota* (Supplementary Figure S3). This is expected given the close relationship of *F. assafoetida* and *D. carota* (Ahmed *et al.* 2005).

Gene ontology (GO) analysis was performed on the whole assembled transcriptome using Blast2GO v1.3.3 (Conesa *et al.* 2005; Götz *et al.* 2008). Blast2GO allowed us to identify similarity of the *F. assafoetida* sequences to GenBank non-redundant proteins (Nr) and Swiss-Prot databases using blastx (E-value <1e^-5^ with the number of hits limited to a maximum of twenty). This was followed by InterProScan search, mapping, and annotation using standard parameters from Blast2GO. After obtaining GO annotation for every transcript, WEGO software (Ye *et al.* 2006) was then used to simplify the output for producing combined graphs for molecular function, cellular process, and biological process. (Supplementary Figure S4). Based on Gene Ontology analyses, a total of 31,524 (52.42%) transcripts had one or more terms assigned.

### Phylogenetic analysis

Protein sequence alignments were generated using CLC bio software (www.Qiagen.com) followed by manual curation. Maximum-likelihood phylogenetic analyses were performed using PhyML version 3.0.1 beta (Anisimova *et al.* 2011) with four rate substitution categories, LG substitution model, BIONJ starting tree and 500 bootstrap repetitions.

### Tissue-specific differential expression analysis

To identify candidate genes for terpenoid and phenylpropanoid metabolism in *F. assafoetida*, we need to compare expression level of genes across different organs. So, the differential expression analysis was utilized to determine how gene expression of target processes differ between different organs.

To determine patterns of gene expression across the different organs, RNA abundance in the assembled transcriptome was quantified using Kallisto (Bray *et al.* 2016), then the differential expression analysis was calculated from 12 samples from UC Davis facility (3 samples from 4 different organs) using the edgeR package in the R statistical environment (FDR <0.05) (Robinson *et al.* 2010b; R Core Team 2016). Sample libraries were normalized by calculating the effective library size and normalization factor using the TMM method on counts data (Robinson and Oshlack 2010a). We then identified genes differentially expressed among plant organs using a generalized linear model (glm) in edgeR and multiple-testing correction via the Benjamini and Hochberg (BH) procedure (Benjamini and Hochberg 1995). The Venn diagram of differential expressed genes of pairwise comparison of different organs was done using Intervene tool (Khan and Mathelier 2017). We provide additional information regarding the experimental design of differential expression analysis in the Supplementary Materials.

### Over-representation analysis of differentially expressed genes

To find significantly enriched GO terms, over-representation analysis (ORA) was done by GOseq package (Young *et al.* 2010), followed by (BH) multiple testing correction to achieve an experiment-wise threshold of *P* < 0.05. Moreover, we investigated the KEGG database by using BlastKOALA (Kanehisa *et al.* 2016) to assign KEGG Orthology (KO) to each transcript. Associated plant-related KEGG pathway ids were obtained from the KO by using KEGGREST Bioconductor package (Tenenbaum 2018). Over-representation analysis of KEGG pathways was also conducted by GOseq package at cut off value p-adjust <0.05 (Young *et al.* 2010) in the R statistical environment (R Core Team 2016). The enriched pathways were visualized with the Pathview Bioconductor package (Luo and Brouwer 2013).

### Gene network analysis (WGCNA)

To find genetic modules that were highly co-expressed across different organs, we performed a weighted gene co-expression network analysis using the WGCNA package v1.63 in R/Bioconductor (Langfelder and Horvath 2008). First, pairwise gene co-expression was calculated from the 12 samples from UC Davis facility (3 samples from 4 different organs) to avoid the possibility of a batch effect that could occur if we included samples sequenced at other facilities.

We further investigated the optimal power for constructing the gene co-expression as indicated in WGCNA best practices and picked the value 12 (Supplementary Figure S5) as a soft power. The network was constructed by setting the type to signed hybrid, minModuleSize to 30, dissimilarity threshold to 0.2, and deepslit to 2.

### Data availability

All transcriptome data were submitted to the NCBI sequence read archive (SRA) with accession number (PRJNA476150) and Temporary Submission ID (SUB4158602). All R scripts for this paper are available at https://github.com/MaloofLab/Amini-G3-2019-Ferula_RNAseq_Analysis. Supplemental material available at Figshare: https://doi.org/10.25387/g3.7609223.

## Results And Discussion

**Figure 1:**
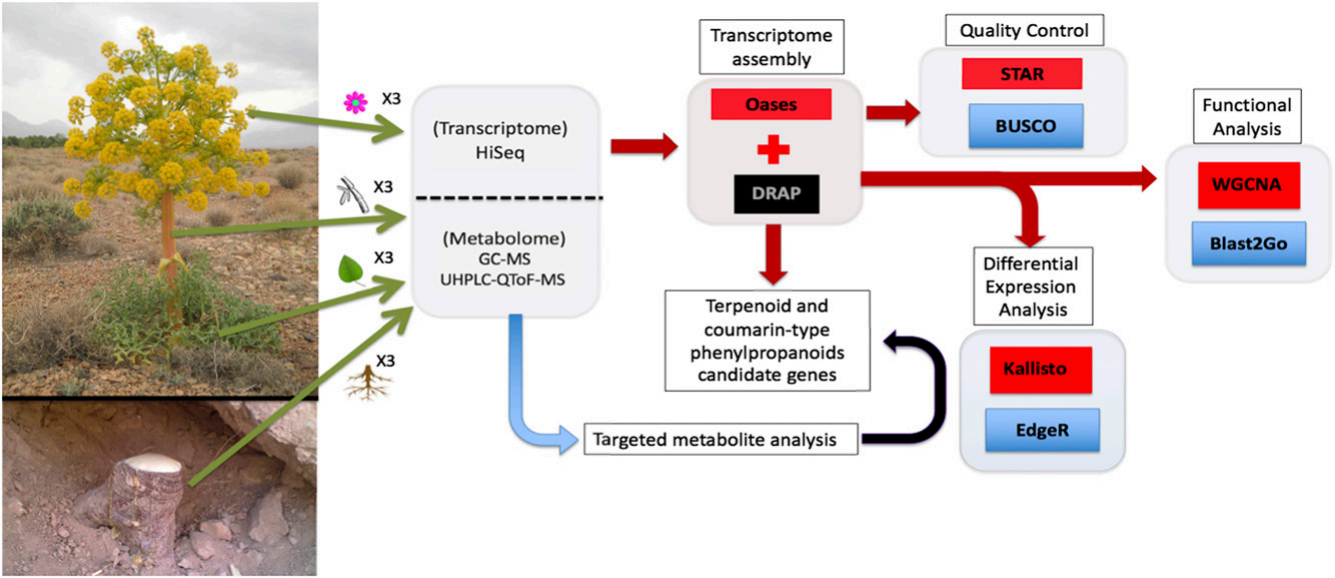
Workflow for transcriptome and metabolite analysis of different organs of *F. assafoetida*. Shown is a schematic overview of the transcriptomics analysis performed on different organs (flowers, leaves, stems, and roots) harvested from three wild *F. assafoetida* plants (green arrows) and used for transcriptome (red) and targeted metabolite analysis (blue). Black arrows highlight the correlation of *de novo* transcriptome and metabolite analysis to identify candidate genes with possible roles in terpenoid and coumarin-type phenylpropanoid metabolism.

To identify the tissue-specific abundance of terpenoid and coumarin-type phenylpropanoid biosynthetic genes we employed correlation studies of gene expression and metabolite abundance across select plant organs (Figure 1).

### Abundance of major oleo-resin metabolites

Coumarin- and flavonoid-type phenylpropanoids, as well as terpenoids are major bioactive constituents of *F. assafoetida*. To investigate the distribution of these metabolites between different organs, we measured the abundance of select terpenoids, flavonoids (luteolin) and coumarins (umbelliferone) using liquid and gas chromatography-mass spectrometry (LC/GC-MS). Among the identified terpenoid metabolites, β-pinene, α-pinene, γ-elemene, β-maaliene, and α/β-eudesmol were the most abundant. Furthermore, terpenoids differed in their abundance in different organs and accumulated at the highest level in roots containing near equal amounts of mono- and sesqui-terpenoids that formed 54% of the resin-derived essential oil (Supplementary Figure S6). This is consistent with previous studies in *D. carota* that suggested that terpenoid metabolism differs substantially between organs (Habegger and Schnitzler 2000).

The coumarin umbelliferone and the flavone luteolin are commonly occurring phenylpropanoid metabolites in members of the Apiaceae family and serve as a precursor for a range of specialized metabolites, including pyrano-coumarins and furano-coumarins (Luo *et al.* 2017; Yao *et al.* 2017). Therefore, we quantified umbelliferone and luteolin across the select plant organs (Supplementary Figure S7A and S7B) and isolated oleo-gum-resin fractions (Supplementary Figure S7C). Umbelliferone and luteolin were most abundant in roots (1.95 ± 0.8 µg/g) and flowers (99.72 ± 40.75 µg/g), respectively (Supplementary Figure S7A and S7B).

### De novo transcriptome assembly and evaluation

Since no reference genome is available for the genus *Ferula*, we performed *de novo* transcriptome assembly to obtain a reference transcriptome assembly as described in the Supplementary Materials. The Oases_DRAP *de novo* transcriptome assembly was used for further analysis.

### Transcriptome annotation

To gain additional insight into biosynthetic pathways all transcripts were queried against the KEGG database with blastKOALA. We found that 43,453 of the 60,134 assembled transcripts were assigned successfully to the KEGG database. We next queried the generated transcriptome data for key genes in terpenoid and phenylpropanoid metabolism. Using homology searches against manually curated protein databases of key gene families with an E-value cut-off of 1e^-75^ (Zerbe *et al.* 2013), we identified 27 candidates for MEV and MEP pathway genes, 32 transcripts with significant matches to terpene synthase (TPS) and triterpene synthase (TTS) genes, and 245 transcripts representing putative P450s. In addition, 142 transcripts with significant matches to phenylpropanoid pathway genes were identified.

### Phylogenetic analysis of transcripts with predicted functions in terpenoid and phenylpropanoid metabolism

Of the identified *F. assafoetida* transcripts, 16 TPS- and TTS-like sequences, as well as 23 putative phenylpropanoid-metabolic enzymes, which represented full-length sequences with the highest similarity to known enzymes were selected for further phylogenetic analysis. To infer possible functions, phylogenetic analysis of these enzyme candidates was performed in comparison to previously reported protein sequences of related Apiaceae species (including *Daucus carota* and *Thapsia garganica*), as well as proteins from Asteraceae and other dicot species that represent key pathway reactions. For clarity, all gene candidates further investigated here have been assigned gene designations based on their predicted function, using common abbreviations for terpenoid- and phenylpropanoid-metabolic enzymes. The corresponding transcript identifiers are given in Supplementary Table S5 and S6.

**Figure 2:**
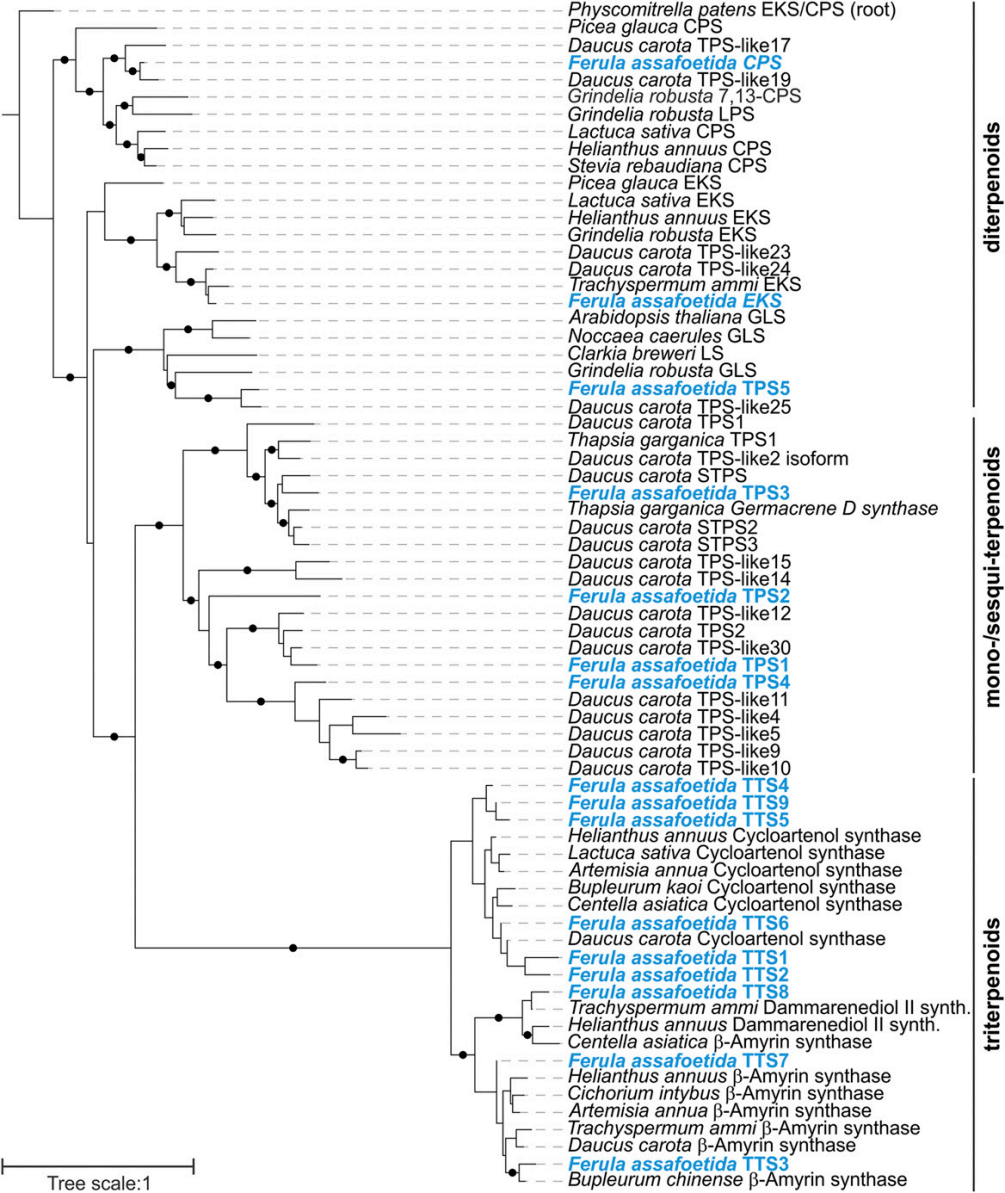
Maximum-likelihood phylogenetic tree of terpene synthase (TPS) and triterpene synthase (TTS) candidates identified in *F. assafoetida* as compared to known enzymes from related plant species. The *Physcomitrella patens* *ent*-kaurene synthase/copalyl diphosphate synthase (EKS/CPS) was used to root the tree. Branches with bootstrap support of >80% (500 repetitions) are highlighted with black dots.

Of the identified TPS-related genes, nine candidates were most closely related to members of the TTS family, including predicted cycloartenol synthases (TTS1-2, 4-6 and 9) with possible roles in sterol biosynthesis, and β-amyrin synthases (TTS3, 7 and 8) putatively involved in the formation of specialized triterpenoid metabolites (Figure 2). In addition, seven candidates were placed within the TPS family. Among these, *F. assafoetida* CPS and EKS clustered with known *ent*-copalyl diphosphate synthases (CPS) and *ent*-kaurene synthases (EKS) with widely conserved functions in gibberellin phytohormone biosynthesis (Peters 2010; Zerbe and Bohlmann 2015), suggesting a similar function. In addition, *F. assafoetida* TPS7 clustered with known geranyllinalool synthases involved in the biosynthesis of defensive homoterpene metabolites (Herde *et al.* 2008; Falara *et al.* 2014; Richter *et al.* 2016). The remaining TPS candidates were placed within the group of mono- and sesqui-TPSs including characterized and predicted TPSs of the close relative *D. carota* (Yahyaa *et al.* 2015; Yahyaa *et al.* 2018). Combined with their phylogenetic relationships, presence of plastidial transit peptides and a characteristic RRX8W motif (Chen *et al.* 2011) suggested a monoterpene synthase function for TPS1 and TPS4, whereas absence of these features indicated a sesquiterpene synthase activity for *F. assafoetida* TPS2 and TPS3. However, an only distant relationship to currently known Apiaceae TPSs, such as the *D. carota* β-caryophyllene synthase DcTPS1 and the geraniol synthase DcTPS2, did not allow a more detailed functional prediction (Keilwagen *et al.* 2017).

**Figure 3:**
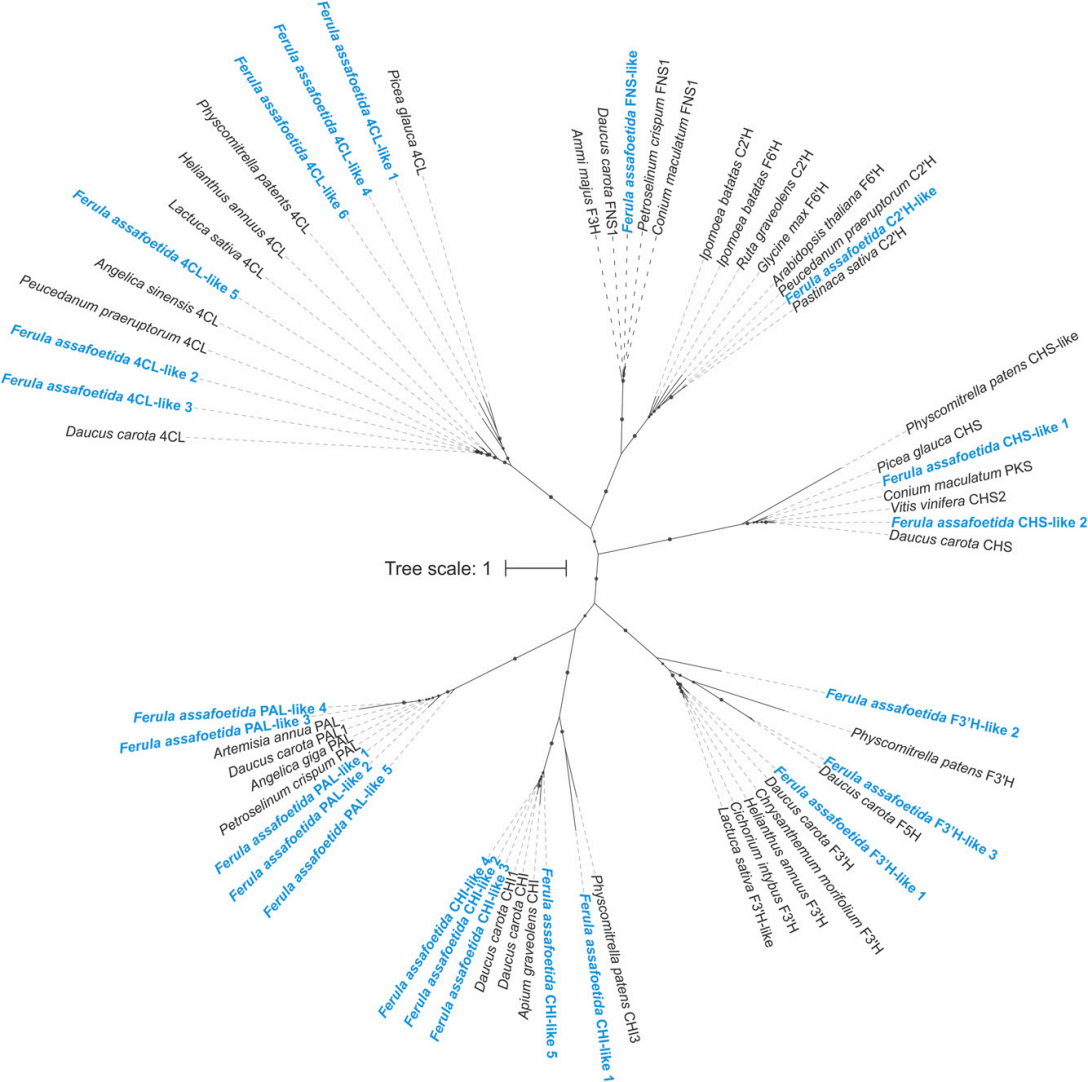
Maximum-likelihood phylogenetic tree illustrating interrelations relationships of enzyme candidates identified in *F. assafoetida* with known phenylpropanoid-biosynthetic enzymes. Branches with bootstrap support of >80% (500 repetitions) are highlighted with black dots. PAL, phenylalanine ammonia-lyase; 4CL, 4-coumarate:CoA-ligase; CHS, chalcone synthases; CHI, chalcone isomerases; FNS, flavone synthases; F3′H, flavanone 3-hydroxylase.

Phylogenetic analysis of candidate genes for coumarin biosynthesis in *F. assafoetida* showed the presence of phenylalanine ammonia-lyase (PAL) and 4-coumarate:CoA-ligase (4CL) as key enzymes controlling phenylpropanoid biosynthesis as multi-gene families of five and six members, respectively (Figure 3). Although the size of the 4CL family in *F. assafoetida* is unclear with 4CL-like 4 and 6 being represented as partial sequences only, 4CL enzymes in other species are more commonly encoded by a single gene (Ehlting *et al.* 1999; Vogt 2010). Hence, a more expansive evolutionary divergence of this pathway component may have occurred in *F. assafoetida*. In contrast, only a single transcript was identified that showed significant similarity to p-coumaroyl-CoA 2′-hydroxylase (C2’H) enzymes that catalyze the hydroxylation of the 4CL product p-coumaroyl-CoA as a key reaction in the formation of coumarins such as umbelliferone (Yao *et al.* 2017) (Figure 3). Unlike coumarins, biosynthesis of flavone metabolites, including luteolin abundant in *F. assafoetida* oleo-gum-resin and other organs (Figure S7), proceeds through the activity of a chalcone synthase (CHS), followed by further modifications by chalcone isomerases (CHI), flavone synthases (FNS), and flavanone 3-hydroxylases (F3′H) (Bourgaud *et al.* 2006; Naoumkina *et al.* 2010; Vogt 2010). Phylogenetic analyses of the identified phenylpropanoid-metabolic gene candidates in *F. assafoetida* showed small gene families of CHS, CHI and F3′H enzymes the occurrence of these pathway enzymes as multi-gene families in other plant species (Naoumkina *et al.* 2010; Vogt 2010). Conversely, FNS appears to be encoded by a single gene with the highest similarity to known type I FNS dioxygenases rather than FNSII enzymes of the CYP93B P450 subfamily in members of the Apiaceae (Britsch 1990; Fliegmann *et al.* 2010).

### Tissue-specific differential expression analysis

The highest levels of differential gene expression were observed between leaves and roots, with a total of 3210 genes differentially expressed (FDR <0.05). Surprisingly, the flower *vs.* root contrast showed substantially fewer genes differentially expressed (420; FDR < 0.05) than leaf *vs.* root; this may be due to greater sample heterogeneity in the flower samples as compared to the leaf samples. The Venn diagram of pairwise comparisons indicated the highest similarly belonged to differential expressed genes of flowers compared to roots and leaves compared to roots (275) (Supplementary Figure S8).

### Over-representation analysis of differentially expressed genes

The differentially expressed genes in different organs are indicative of biological function most relevant to each organ. Thus, we investigated the GO term over-representation of differentially expressed genes among select pairwise organ comparisons (Supplementary Figure S9). Photosynthesis GO terms were significantly upregulated in leaves (p-adjust <1e^-09^) and flowers (p-adjust <4e^-05^) as compared to roots (Figure S9A and S9B). With respect to terpenoid we observed terpene synthesis activity GO term as over-represented in up-regulated genes in roots *vs.* flowers (p-adjust <0.007) and up-regulated in flowers *vs.* stems (p-adjust <0.0002), suggesting that roots have the highest expression of the GO category, followed by flowers, and then stems (Figure S9C).

To examine the tissue-specificity of the biosynthetic pathways of the metabolites targeted here, we further studied the over-represented KEGG orthology (KO) terms in differentially expressed genes using GOseq package. The pathway analysis using KEGG database indicated several over-represented KO terms for the differential expressed genes in various organs (Supplementary Figure S10). We found that the KEGG pathway for biosynthesis of sesqui- and tri-terpenoid was upregulated in flowers *vs.* stems while the KO term for flavonoid biosynthesis was upregulated in flowers *vs.* other organs (Supplementary Figure S10). These two pathways were visualized, and the candidate genes were colored (Supplementary Figure S11 and S12).

### Gene network analysis (WGCNA)

An alternative to identifying gene involved in terpenoid and flavonoid biosynthesis is to use gene co-expression to reconstruct genetic modules. Because this method treats each sample separately it may be better at identifying clusters of genes that function together. To accomplish this, we performed a weighted gene co-expression network analysis (WGCNA) (Langfelder and Horvath 2008) to find genetic modules which are highly co-expressed across the 12 samples (3 samples from 4 different organs) in *F. assafoetida*. WGCNA found 43 non-overlapping modules ranging from 38 to 3557 total gene size (Supplementary Figure S13).

Our metabolite analysis had indicated substantial variation in terpenoid and flavonoid abundance among different organs (Supplementary Figure S6 and S7). To find modules associated with these patterns, we next asked if any module could be related to our metabolites of interest. To do this, we calculated the correlation of each module’s eigengene expression value and the measured metabolites of interest: terpenoid and coumarin-type phenylpropanoids including umbelliferone and luteolin.

One module of interest is the darkseagreen3 module. The darkseagreen3 module eigengene had significant correlations with sesquiterpenoid (r= -0.76 and adjusted p–value <0.0038) and the luteolin flavone compound (r= 0.83 and adjusted p–value <0.0014). Furthermore, the GO term significantly associated with the darkseagreen3 module was terpene synthase activity (Supplementary Figure S14A). Combined with the correlation with sesquiterpenoid content (adjusted p–value <0.0038), it is clear that genes in this module are important for sesquiterpenoid biosynthesis. The correlation coefficient (r= -0.76) indicated negative correlations between the darkseagreen3 module eigengene and sesquiterpenoid, meaning that higher expression of this module corresponds to less sesquiterpene synthesis. This suggests that the darkseagreen3 module contains genes which act as repressors for sesquiterpene synthesis or that shunt precursors into alternative pathways.

The darkseagreen3 module also was over-represented in GO terms “transferase activity, transferring hexosyl groups” that are parent of the “Luteolinidin 5-O-glucosyltransferase activity” term. This suggests that the darkseagreen3 module could be related to luteolin biosynthesis (Figure S14A). Since the flowers had the highest level of Luteolin while roots had the highest level of sesquiterpenoid, it could be concluded the darkseagreen3 module regulating the balance between luteolin and sesquiterpene biosynthesis.

**Figure 4:**
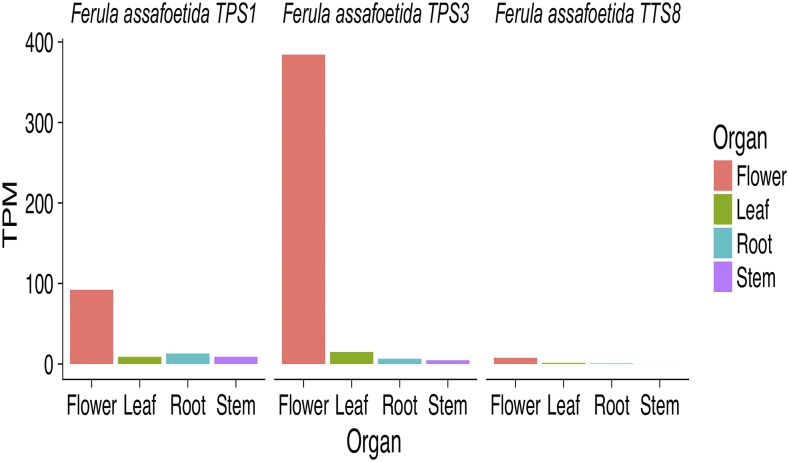
The TPM (Transcripts Per Kilobase Million values) of the candidate genes of *TPS*, terpene synthases; *TTS*, triterpenoid metabolism for different organs. The candidate gene names were provided in Supplementary Table S5.

Having identified the darkseagreen3 module as being associated with sesquiterpenoid and flavonoid biosynthesis we next investigated the differential expression patterns of the identified candidate genes for terpenoid and flavonoid metabolism. Note that some of these candidate genes had very low expression abundance and were not considered in differential expression analysis. Among the candidate genes of the terpenoid pathway, *F. TPS1*, *TPS3*, and *TTS8*, were located in darkseagreen3 module. These candidate genes of terpenoid pathway, were more abundant in flowers as compared to all other organs tested (Figure 4, Supplementary Figure S11 and Supplementary Table S7). This observation was consistent with the eigengene values for upregulated sesqui- and tri-terpenoid biosynthetic pathways among different organs (Figure S14B). Thus, these are candidate genes for the high levels of sesqui- and tri-terpenoid compounds observed in flowers.

**Figure 5:**
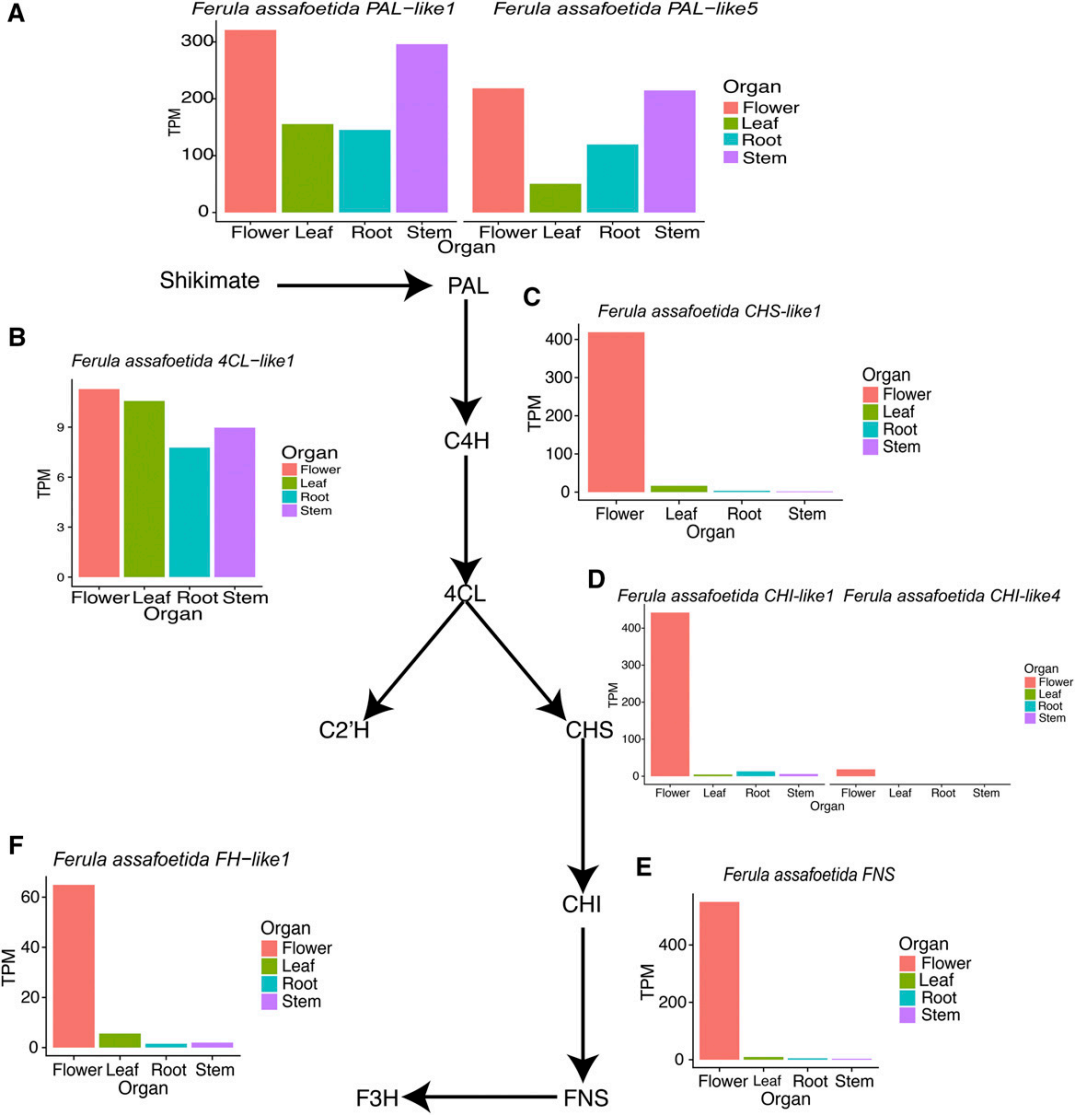
The TPM (Transcripts Per Kilobase Million values) of the candidate genes of *PAL* (A), *4CL* (B), *CHS* (C), *CHI* (D), *FNS* (E), *F3H* (F) as key genes of phenylalanine reactions for different organs. *PAL*, phenylalanine ammonia-lyase; *4CL*, 4-coumarate:CoA-ligase; *CHS*, chalcone synthases; *CHI*, chalcone isomerases; *FNS*, flavone synthases; and *F3′H*, flavanone 3-hydroxylase. The candidate gene names were provided in Supplementary Table S6.

Consistent with luteolin being most abundant in flowers (Supplementary Figure S7B) (Naoumkina *et al.* 2010; Vogt 2010; Li *et al.* 2018), we identified several candidate genes including *F. CHI-like1*, *CHI-like4*, *CHS-like1*, *FH-like1*, and *FNS* with predicted roles in phenylpropanoid metabolism that were most abundant in flowers and were also located in the darkseagreen3 WGCNA module (Figure 5). Note that, *F. assafoetida CHI-like4*, *FH-like1*, and *FNS* were upregulated in flavonoid biosynthesis pathway (Supplementary Figure S12). FNS activity is essential for luteolin biosynthesis (Chen *et al.* 2018), thus, *FNS* could be considered as candidate gene for luteolin biosynthesis in *F. assafoetida*. All these candidates were upregulated in flowers (Figure 5, Supplementary Figure S12 and Supplementary Table S8).

Also of interest, is the coral module. The coral module has a positive and significant correlation (r = 0.72, adjusted p-value = 0.008) with sesquiterpene. The coral module exhibited higher abundance in roots than other organ types. This means that the genes in coral module may act as activators for sesquiterpene synthesis. See Figure S15 for a depiction of how the darkseagreen3 and coral modules may act in sesquiterpene biosynthesis. The coral module included three transcripts, “oases6_k43_Locus_7389_Transcript_8_1”, “oases6_k31_Locus_24182_Transcript_3_1”, and “oases6_CL10402Contig1_1”, that had significant matches to terpene synthase genes. These three genes also have a GO term of terpene synthase activity indicating their contribution to this function. Furthermore, these candidate genes all had a higher expression level in roots *vs.* flowers (Figure S16).

Combining tissue-specific transcriptome and metabolite analyses of the medicinal plant *F. assafoetida* identified candidate genes with possible roles in the biosynthesis of sesquiterpenoid and flavonoid metabolites as major bioactive constituents in the plants oleo-gum-resin. These resources can facilitate further gene function studies toward key bioactive natural products that define the medicinal properties of this traditional medicinal plant. Moreover, we provide detailed assembly protocol to enable efficient transcriptome analyses in a broader range of non-model plant species.
